# Concomitant Medication Effects on Immune Checkpoint Inhibitor Efficacy and Toxicity

**DOI:** 10.3389/fonc.2022.836934

**Published:** 2022-05-25

**Authors:** Brendan Sieber, Julius Strauss, Zihai Li, Margaret E. Gatti-Mays

**Affiliations:** ^1^ School of Medicine, The Ohio State University, Columbus, OH, United States; ^2^ The Laboratory of Tumor Immunology and Biology, National Cancer Institute, National Institutes of Health, Bethesda, MD, United States; ^3^ Division of Medical Oncology, The Ohio State University, Columbus, OH, United States; ^4^ Pelotonia Institute for Immuno-Oncology, The Ohio State University, Columbus, OH, United States

**Keywords:** immune checkpoint inhibitor (ICI), metformin, ACE (angiotensin coverting enzyme inhibitor) inhibitor, ARB (angiotensin receptor blocker), aspirin

## Abstract

There are multiple approved indications for immune checkpoint inhibitors (ICI) in patients with advanced solid tumors. Polypharmacy, defined as the use of ≥ 5 medications, is common among cancer patients. The impact of these non-oncologic medications on ICI efficacy or the development of side effects, specifically immune related adverse events (irAEs), is unclear. Recent clinical studies investigating the connection between concomitant medications and ICI efficacy have produced conflicting results. A systematic literature search was performed on PubMed to identify published clinical studies evaluating the impact of metformin, angiotensin-converting-enzyme inhibitor (ACEi), angiotensin receptor blockers (ARBs) and aspirin on ICI outcomes and toxicity in patients with advanced solid tumors. Clinical outcomes assessed included overall response rate, progression free survival, overall patient survival and the development of adverse events, specifically irAEs. A total of 10 retrospective studies were identified. Most studies reported a small percentage (range 8% to 42%) of their study population taking the concomitant medications of interest. Collectively, the studies did not identify a significant impact on ICI efficacy with concomitant medication use. In addition, the impact on irAEs was rarely reported in these studies but no significant group effect on reported toxicities or irAEs was found. This review provides a comprehensive analysis of current clinical studies and illustrates potential alterations in the tumor microenvironment induced by the medications. Given the high occurrence of polypharmacy among patients with advanced cancer, gaining a better understanding of the impact of non-oncologic medications on immunotherapy is necessary to improve ICI efficacy and reduce toxicity.

## Introduction

The approval of the first immune checkpoint inhibitor (ICI) ipilimumab in 2011 represented significant progress in melanoma with a decrease in the overall death rate by 7% per year between 2013 and 2017 (1). The full impact of ipilimumab was seen years later when there was a 2.2% decline in deaths between 2016 and 2017 – the largest single year decline in the U.S. cancer death rate seen at that time (1). U.S. FDA approved ICIs include: anti-programmed cell death protein 1 (PD-1; nivolumab, pembrolizumab, cemiplimab, dostarlimab), anti-programmed death-ligand 1 (PD-L1; atezolizumab, avelumab, and durvalumab), and anti-cytotoxic T-lymphocyte associated protein 4 (CTLA4; ipilimumab) monoclonal antibodies. Several meta-analyses and systemic reviews have demonstrated improved clinical outcomes, improved health-related quality of life, improved patient reported outcomes and better tolerance in patients who receive immunotherapy +/- chemotherapy compared to chemotherapy (2–4). With widespread acceptance of ICIs into standard practice, a greater understanding of the impact of baseline medications and comorbidities is needed.

Oncology patients are frequently prescribed multiple medications for preexisting comorbidities or side effects from treatment. Polypharmacy, defined as five or more prescriptions (5), is common among cancer patients with one study reporting polypharmacy in up to 84% of patients (6). Given the high prevalence of polypharmacy and the potential for interactions among concomitant medications and ICIs, a growing number of researchers have focused on the potential interactions of common medications and ICIs. Several investigators have found that patients taking concomitant medications like proton pump inhibitors, antibiotics or steroids while receiving immunotherapy had less clinical benefit than patients who were taking these concomitant medications while receiving chemotherapy, suggesting a larger impact of concomitant medications on ICI efficacy than standard chemotherapy (7, 8).

ICIs engage the immune system to eradicate tumor cells through a variety of mechanisms and pathways. Therefore, potential interactions of concomitant medication and ICIs go well beyond commonly evaluated drug-drug interactions (e.g., CYP450 inducers or inhibitors) to include modulation of the tumor microenvironment (TME) through engagement of the peripheral immune response or alterations in the normal flora composition (9–11). In addition, polypharmacy is linked to decreased medication adherence (6), representing another obstacle for optimal management of oncology patients with chronic diseases and durable responses to ICIs.

Among the 15 most commonly prescribed medications in the U.S (12)., were the following medications of interest for TME modulation: lisinopril (#1), metformin (#4), and losartan (#12). In addition, aspirin is routinely used by more than 29 million people in the US (13) due to its well established anti-inflammatory effects. Recently, the anti-platelet activity of aspirin has been linked to promoting anti-tumor T cell immunity (14–16). Given the prevalence of these medications and their potential immune modulating roles, we investigated if concurrent use of metformin, ACEi/ARBs or aspirin impacted ICI efficacy as a primary outcome and if toxicity was affected, specifically irAEs as a secondary outcome.

### Metformin

There has been great interest in the role of metformin in cancer treatment (17) as well as in prevention (18) long before the use of ICIs. Metformin is a biguanide and is a commonly used anti-diabetic medication (#4). Preclinical studies have shown that in addition to increased insulin sensitivity and reduced gluconeogenesis, metformin inhibits cellular growth *via* regulation of AMP-activated protein kinase (AMPK) and liver kinase B1 (LKB1) pathways, leading to inhibition of the rapamycin (mTOR) pathway (19–21). There is growing data supporting that metformin also effects the TME and renders it more receptive for immunotherapy through the modulation of immune cells including T cells, natural killer (NK) cells, myeloid-derived suppressor cells (MDSCs) and tumor associated macrophages (TAMs) (20, 22–24). However, metformin also inhibits PD-L1 expression on cancer cells through the action of AMPK (25–27) leading to concerns that concurrent metformin use may dampen the effect of ICIs. Given the high prevalence of metformin use, the clinical impact of this medication on ICI toxicity is of great interest. Several clinical trials have evaluated are prospectively evaluating the impact of metformin in combination with ICIs [NCT03048500, NCT03800602] with highly anticipated results.

### ACEi/ARBs

ACEi (Lisinopril # 1) and ARBs (Losartan # 12) are commonly used medications for patients with cardiovascular disorders and diabetes (28). The main mechanism by which ACEi/ARBs act is through reducing angiotensin (AT) II by blocking AT I receptors [AT1R] (receptors found on heart, blood vessels, and kidneys). This decrease in AT II induced by ACEi/ARBs may assist in the growth of tumor cells because AT II binds to angiotension II receptors (AT2R) which promotes cellular proliferation and activates intracellular kinases including EGFR and VEGF. This ultimately leads to increased tumor plasticity and therapeutic resistance (29–31). Furthermore, the use of ACEi/ARBs may alter the TME through activating CD4 T cells, NK cells, and altering immunocytokines (28, 32, 33). However, some have demonstrated negative effects on the TME including enhanced M2 differentiation and activated mast cells, raising concerns about concurrent ACEi/ARB and ICI use (31, 32).

### Aspirin

Aspirin, a well-known inhibitor of prostaglandin synthesis, is widely used for its anti-inflammatory properties which may mediate effects of immunotherapy. Aspirin inhibits prostaglandin E2 (PGE2) which reduces pro-inflammatory cytokines (34) and reduces cyclooxygenase 2 which decreases TAMs, MDSC, and PD-L1 expression (35). Several clinical trials are evaluating the impact of concurrent aspirin use on ICI efficacy in solid tumors [NCT04188119, NCT03245489]. We eagerly await these results.

## Methods

In this literature review, we focused on retrospective clinical studies which included: 1) adult patients (≥18 years old), 2) with an advanced solid tumor, 3) who received an FDA approved ICI including anti-PD1 (nivolumab, pembrolizumab, cemiplimab), anti-PD-L1 (atezolizumab, avelumab, and durvalumab), or anti-CTLA4 (ipilimumab) therapies, and 4) taking metformin, ACEi, ARB, or aspirin at baseline. Patients taking ICIs in combination with chemotherapy or radiation therapy were excluded to help isolate the impact of the concomitant medication. PubMed was searched on June 1, 2021 using the following search terms: ((“Angiotensin-Converting Enzyme Inhibitors”[Mesh] OR “ACE Inhibitors” OR “Angiotensin II Type 2 Receptor Blockers”[Mesh] OR “Metformin”[Mesh] OR “Metformin” OR “Aspirin”[Mesh] OR “Aspirin”) AND (“Immune Checkpoint Inhibitors”[Mesh] OR “Immune Checkpoint Inhibitors” OR “anti-cytotoxic T-lymphocyte associated protein-4” OR “anti-programmed death protein 1” OR “anti-programmed death ligand 1”)).

Included studies reported patient outcomes per RECISTv1.1 (36) with overall response rate (ORR; defined as complete responses plus partial responses divided by the total patient population), progression free survival (PFS; defined as the length of time from starting a therapy until disease progression), and overall survival (OS; defined as the length of time from starting ICI therapy until death). The impact of toxicity was also reviewed and irAEs extracted to evaluate the secondary objective.

## Results

The PubMed search returned 45 manuscripts. The cited primary papers in the review manuscripts identified were extracted and included in the initial review after duplicates were removed. A total of 69 primary research studies were identified; 51 full-text articles were assessed for eligibility of which 10 retrospective studies met the inclusion criteria for this review (**Table 1** and **Supplemental Figure 1**). Manuscripts were reviewed independently by B.S. and M.G.M. with any conflicting manuscripts reviewed jointly. Reasons for exclusion were insufficient clinical outcomes data (n =22), combination immunotherapy-chemotherapy (n = 10), absence of selected concomitant medications (n = 7) or hematologic malignancy (n =2).

**Table 1:** Retrospective clinical studies evaluating effect of metformin, ACEi/ARBs or aspirin on ICI efficacy and toxicity in patients with advanced solid sumors.

Study Information (Reference No.)	Study Description	Tumor Type	ICI Used	Concomitant Medications *(n, %)*
Afzal et al., 2018 (20)	Retrospective study ICI efficacy irAE development n=55	Melanoma (n=55)	Ipilimumab Nivolumab Pembrolizumab	Metformin (22, 40.0%)
Afzal et al., 2019 (24)	Retrospective study ICI efficacy irAE development n=50	NSCLC (n=50)	Pembrolizumab Nivolumab Atezolizumab	Metformin (21, 42.0%)
Cortellini et al., 2020 (37)	Retrospective study ICI efficacy n=1012	NSCLC (n=528) Melanoma (n=263) RCC (n=185) Others (n = 36)	Pembrolizumab Nivolumab Atezolizumab	Metformin (114, 11.3%) ACEi/ARB (313, 30.9%) Aspirin (189, 18.7%)
Cortellini et al., 2021 (8)	Retrospective study ICI efficacy n=950	NSCLC (n=950)	Pembrolizumab	Metformin (125, 13.2%) Aspirin (254, 26.8%)
Failing et al., 2016 (38)	Retrospective Study ICI efficacy irAE development n=159	Melanoma (n=159)	Ipilimumab	Metformin (12, 8%) ACEi/ARB (30, 18.9%) Aspirin (38, 23.9%)
Gandhi et al., 2020 (39)	Retrospective study ICI efficacy irAE development n=210	Melanoma (n=101) Lung (n=68) Renal (n=22) Bladder (n=10) Other (n = 9)	Nivolumab Pembrolizumab Ipilimumab	Metformin (23, 11.0%) Aspirin (58, 27.6%)
Gaucher et al., 2021 (40)	Retrospective study ICI efficacy n=372	Lung (n=166), Melanoma (n=110) Genitourinary (n=27) Head and neck (n=48) Other (n = 21)	Ipilimumab Nivolumab Pembrolizumab Ipilimumab + Nivolumab	Metformin (17, 4.6%)
Medjebar et al., 2020 (31)	Retrospective study ICI efficacy n=168	NSCLC (n=178)	Pembrolizumab Nivolumab Durvalumab	ACEi (22, 13.1%)
Svaton et al., 2020 (35)	Retrospective study ICI efficacy n=224	NSCLC (n=224)	Nivolumab	Metformin (18; 8.0%)
Tozuka et al., 2021 (32)	Retrospective study ICI efficacy n=256	NSCLC (n=256)	Nivolumab Pembrolizumab Atezolizumab	ACEi/ARBs (37, 14.4%)

All of the identified retrospective studies involved patients with advanced solid tumors (**Table 2** and **Supplemental Table 1**). There were no studies in the neoadjuvant or adjuvant settings. Given that ICI efficacy was the primary outcome of this review, all 10 studies included data on efficacy of ICI monotherapy with concurrent medication use. However, only 4 of the retrospective studies included data on the effect of concurrent medications on toxicity and irAEs. Furthermore, despite sizable study populations for the identified retrospective studies, only a small proportion of patients in each study were taking the concomitant medication of interest (range 8% to 42%, with most reporting 10-20% of patients taking a concomitant medication; **Table 1**).

**Table 2:** Summary of impact of concomitant medications on ICI efficacy and irAE development.

Study (Reference)	ICI used	Median OS	Median PFS	ORR (%)		irAE
**Metformin**						
Afzal et al., 2018 (20)	Ipilimumab, Nivolumab, and/or Pembrolizumab	**↔**	**↔**	**↔**		**↔** overall ↑ pneumonitis
Afzal et al., 2019 (24)	Pembrolizumab, Nivolumab, or Atezolizumab	**↔**	**↔**	**↔**		**↔ overall** ↓ pneumonitis
Cortellini et al., 2020 (37)	Pembrolizumab, Nivolumab, or Atezolizumab	↔*	**↔**	**↔**		n/r
Cortellini et al., 2021 (8)	Pembrolizumab	**↔**	**↔**	**↔**		n/r
Failing et al., 2016 (38)	Ipilimumab	**↔**	**↔**	**↔**		**↔**
Gandhi et al., 2020 (39)	Nivolumab, Pembrolizumab, or Ipilimumab	**↔**	**↔**	n/r		n/r per medication
Gaucher et al., 2021 (40)	Ipilimumab, Nivolumab, Pembrolizumab, or Ipilimumab/Nivolumab	**↔**	n/r	**↑**		n/r
Svaton et al., 2020 (35)	Nivolumab	**↔**	**↔**	n/r		n/r
**ACEi/ARBs**						
Cortellini et al., 2020 (37)	Pembrolizumab, Nivolumab, or Atezolizumab	**↔**	**↔**	↔*		n/r
Failing et al., 2016 (38)	Ipilimumab	**↔**	**↔**	**↔**		**↔**
Medjebar et al., 2020 (31)	Pembrolizumab, Nivolumab or Durvalumab	↓*	↓*	n/r		n/r
Tozuka et al., 2021 (32)	Nivolumab, Pembrolizumab, and Atezolizumab	**↔**	**↑**	n/r		n/r
**Aspirin**						
Cortellini et al., 2020 (37)	Pembrolizumab, Nivolumab, or Atezolizumab	**↔**	*↑	*↑		n/r
Cortellini et al., 2021 (8)	Pembrolizumab	**↔**	**↔**	**↔**		n/r
Failing, et al., 2016 (38)	Ipilimumab	**↔**	**↔**	**↔**		**↔**
Gandhi et al., 2020 (39)	Nivolumab, Pembrolizumab, or Ipilimumab	**↔**	**↔**	n/r		n/r per medication

↑= statistically significant positive impact on ICI efficacy with concomitant medication use; ↓ = statistically significant negative impact with concomitant medication use, ↔ = no association between ICI effect/toxicity and concomitant medication use association between ICI efficacy and concomitant medication use. * = adjusted ratio with significance. n/r , not reported. See **Supplemental Table 1** for full study details.

### Metformin

Eight studies evaluated the effect of metformin on ICI efficacy with three studies specifically detailing toxicity (**Table 2**, **Supplemental Table 1**). Two studies analyzed the effects in patients with melanoma [Afzal et al. (20) and Failing et al. (38)], three analyzed the effects in patients with non-small cell lung cancer [NSCLC; Afzal et al. (24), Svaton et al., 2020 (35), and Cortellini et al. (8)], and three analyzed patients with solid tumors [Gandhi et al. (39), Gaucher et al. (40), and Cortellini et al., (37)]. Only Gaucher et al. reported a significant improvement in clinical response in patients taking metformin plus ICI therapy (ORR = 47.1% with *vs*. 24.5% without, p = 0.02) but this benefit did not equate to longer OS or PFS (40). Three studies reported a trend towards improved ICI efficacy with metformin but was not statistically significant (8, 20, 24). In addition, three studies reported a trend towards worse patient outcomes with metformin use during ICI treatment but again none were statistically significant (35, 38, 39). Within these 8 retrospective studies, no clear trend on the impact of metformin on ICI efficacy was observed.

Metformin did not worsen toxicities or irAE development in the three studies where it was reported (20, 24). However, pneumonitis was slightly lower among patients with NSCLC who took metformin (24) and higher among patients with melanoma who took metformin (20).

### ACEi/ARBs

Four retrospective studies evaluated the effect of ACEi/ARBs on ICI efficacy with only one study reporting data on toxicity (**Table 2** and **Supplemental Table 1**). Cortellini et al. (37) evaluated the effect of ACEi/ARBs in patients with solid tumors, while Failing et al. (38) studied patients with melanoma and Medjebar et al. (31) and Tozuka et al. (32), evaluated the effect on NSCLC.

Medjebar et al. found worse outcomes in melanoma patients who used ACEi (OS = 9.82 with *vs*. 11.6 months without; HR = 1.6, p=0.07 and PFS = 1.97 months with *vs*. 2.56 months without; HR = 1.8, p=0.01) but this PFS difference of less than 1 month is likely not clinically significant (31). Tozuka et al., 2021 reported better outcomes with a significant increase in PFS with concurrent ACEi/ARBs plus ICI (PFS = 6.0 months with *vs*. 2.2 months without; HR = 0.59, p=0.008), and while there was a trend towards OS improvement, statistical significance was not achieved (OS = 22.6 months with *vs*. 14.7 months without; HR = 0.71, p=0.131) (32). The remaining studies reported a non-significant trend towards improved ICI efficacy with ARB use [Failing et al., 2016 (38): ORR = 38% with *vs* 28% without; OR = 2.01, p=0.22; Cortellini et al., 2020 (37): ORR = 42.9% with *vs*. 35.3% without; aOR = 1.26, p = 0.12)]. Only Failing and colleagues reported the effect of ACEi/ARBs on toxicity and found no impact of ACEi/ARBs on toxicity/irAEs (HR 1.60, p = 0.37) (38).

### Aspirin

Four studies evaluated the effect of the anti-inflammatory aspirin on ICI efficacy (**Table 2** and **Supplemental Table 1**). Cortellini et al. (37) and Gandhi et al. (39) evaluated patients with solid tumors, while Failing et al. (38) evaluated aspirin in patients with melanoma. Cortellini et al. (8) analyzed the effect of aspirin in patients with NSCLC. When adjusted for functional status, metastatic burden and body mass index, Cortellini et al., found a significant improvement in ICI efficacy in patients with baseline aspirin use (ORR = 44.4% with *vs*. 36.0% without; aHR 1.47, p = 0.03) (37) which was not supported by the other three studies (8, 38, 39). Again, Failing and colleagues was the only study which evaluated the effect of aspirin on toxicity. There was no increase in side effects with the use of aspirin but there was a nonsignificant trend towards greater toxicity with concurrent ICI and aspirin use (41% with *vs* 25% without; OR 2.19, p = 0.10).

## Discussion

In this systematic review, we found that despite theoretical concerns about the impact of metformin, ACEi/ARBs and aspirin on ICI efficacy and toxicities, most published retrospective clinical studies did not find a significant association. There are several ongoing clinical trials prospectively evaluating the impact of metformin or aspirin on ICI efficacy and we eagerly await their results. However, until these studies report out, the question of how best to counsel patients on potential drug-TME interactions and specifically if these commonly used medication may help to enhance immunotherapy responses remains.

While Gaucher et al. reported significantly improved ORR in patients using metformin (47.1% with *vs* 24.5% without), this did not translate to improved OS (40). Furthermore, only 17 patients in their cohort of 372 (4.6%) reported baseline metformin use. The remaining studies which evaluated metformin did not find a significant impact (positive or negative) on ICI efficacy. Despite promising preclinical data, clinical studies have not supported that metformin significantly impacts tumor growth or the TME.

Studies which evaluated concurrent ACEi/ARB use also produced conflicting results with Tozuka et al. reporting concurrent ACEi/ARB use improved PFS (6.0 months with *vs*. 2.2 months without; p = 0.01) while Medjebar et al. reporting that concurrent ACEi use statistically worsened both PFS (Δ – 0.6 months) and OS (Δ – 1.8 months) in their study population. In both studies, fewer than 15% of patients reported taking an ACEi/ARB at baseline which raises concerns about power. In addition, while the PFS and OS were statistically worse in the study by Medjebar et al, this small difference is likely not clinically significant. Overall, ACEi/ARBs as a group do not appear to have a significant impact on ICI efficacy or toxicity but there may be differing effects of ACEi compared to ARBs. A retrospective study by Strauss et al. presented at EORTC-NCI-AACR 2020 (28) in 597 pts with a wide array of solid tumors showed that concomitant ARB use was associated with significantly improved ORR (33.8% *vs* 17.0%, p = 0.001) and improved median OS (35.2 *vs* 18.8 months, p=0.006) in patients receiving ICI while concomitant ACEi use was not associated with any improvement in ORR (19.5% *vs* 17.0%, p=0.6) or median OS (26.2 *vs* 18.8, p = 0.078). Similar findings were seen in a retrospective study by Pereira, et al. presented at ESMO 2021 which looked at 127 pts with NSCLC receiving ICI therapy and which showed that ARB use was associated with an increase in ORR (26.7%), in contrast to ACEi use was not associated with ORR improvement (12.5%) compared to patients receiving neither agent (14.9%) (41). The ARB group had a prolonged median PFS and OS compared to the control group (HR 0.401, 95%CI 0.174-0.929 and HR 0.438, 95%CI 0.189-1.015, respectively) while no statistically impact in PFS (HR 0.872, 95%CI 0.460-1.654) or OS (HR 0.747, 95%CI 0.393-1.419) was observed in ACEi group compared with control group. This discrepancy may be due to the fact that ARBs selectively target AT1R while ACEi target AT1R and AT2R and these two receptors may have different TME effects.

Of the four concomitant medications evaluated in this systematic review, aspirin was the most commonly used with studies reporting 20-25% of patients with baseline use. Concurrent aspirin administration may improve ORR and PFS in patients on ICI monotherapies as suggested by Cortellini et al. who reported improved ORR and PFS after adjusting for functional status, metastatic burden and body mass index (8). The other studies did not detect any difference in ICI efficacy with aspirin use.

Most of these retrospective studies focused on clinical efficacy (OS, PFS and ORR). However, given that many ICIs can produce deep and durable clinical responses, the impact on toxicity should also be considered. Furthermore, some toxicities (i.e., diarrhea) are overlapping between concomitant medications like metformin and ICIs and it is unclear if co-administration increase the risk of specific irAEs. Only 4 of the 10 studies reported toxicity data. No significant associations were found with concomitant medication use and ICI monotherapy. However, it is interesting to note that in patients with NSCLC who received metformin and ICI monotherapy, the risk of developing pneumonitis was lower than in patients who were not taking metformin [4.8% with *vs* 17.2% without, p = 0.10 (24)]. Given that patients with NSCLC have the highest risk of developing ICI-related pneumonitis (42), metformin may help to reduce this risk in this patient population but may not benefit all solid tumor patients as there was a higher risk of pneumonitis reported in melanoma patients who took metformin and ICIs than those patients who did not take metformin (20). In both of these retrospective studies only 40% of their relatively small population reported metformin use and the occurrence of pneumonitis was limited to only a few patients. Again, prospective data is greatly needed to answer the question how baseline medications impact toxicity and irAEs.

This systematic review has several limitations. First, studies included in this review were limited to only those published at the time of the search. Abstracts presented at conferences were excluded as these are not yet peer reviewed. Focusing on peer reviewed manuscripts may have create publication bias. Second, we focused on the most commonly used medications of interest in our patient population. Prior research has evaluated the impact of concomitant medications that alter the normal flora [i.e, antibiotics, proton pump inhibitors, probiotics (10, 43)] as well as known immune modulators [prednisone (44–46)]. Given the plethora of published data, the review was focused away from these agents. However, it is likely that patients in these retrospective studies were taking more than one concomitant medication. It is impossible to fully account for these effects but is important to note given the discordant findings of some of the studies. Third, many of these retrospective studies had very small sample sizes and were likely underpowered to evaluated fully investigate these associations but the detected effects are hypothesis generating.

Given the high prevalence of polypharmacy, a better understanding of the impact of baseline medications on ICI efficacy and toxicity is greatly needed. This systemic review highlights the ongoing knowledge gaps about the true impact of concomitant medications on ICI efficacy but specifically on immune related toxicity. Patients should be educated that to date, no specific associations have been identified with worse clinical outcomes or higher frequency of toxicity in patients who are taking metformin, ACEi/ARBs or aspirin while being treated with immunotherapy. However, the current literature review highlights the need for evaluation of concomitant medications among different tumor types. As we have highlighted here a medication like metformin may help reduce pneumonitis in NSCLC but may increase the risk in melanoma. Prospective studies and randomized clinical trials are ongoing and are greatly needed to properly evaluate the impact of these medications on immunotherapy cancer treatments, specifically the impact of these medications on the TME. In addition, evolving real world data over the next 5 to 10 years of patients who receive ICIs as part of standard of care treatment will greatly contribute to these knowledge gaps. Finally, most of the published studies to date have evaluated the impact of concomitant medications on efficacy, but few have evaluated their potential impact on irAEs or quality of life. As patients who receive ICIs live longer with stable or regressing disease, we must focus on the impact of concomitant medications in irAE development and general ICI tolerability. This is a great unmet need in immunotherapy as some low grade side effects can last for years.

## Conclusion

In this systematic review, concomitant use of metformin, ACEi/ARB or aspirin with ICI monotherapy in patients with advanced solid tumors did not significantly impact ICI efficacy or worsen toxicity. However, most studies to date are small and underpowered. Larger, prospective studies are greatly needed to address this unmet need and identify whether specific medications may be preferred for the treatment of non-oncologic conditions during cancer therapy.

## Author Contributions

The study hypothesis was generated by BS, JS, and MGM. Study design was generated by BS and MGM. Both JS and ZL provided expert feedback on study design. BS and MGM analyzed the data. All authors contributed to the article and approved the submitted version.

## Funding

This work was supported by The Ohio State University College of Medicine – Samuel J. Roessler Memorial Scholarship (BS), Columbus, OH, USA and supported by The Ohio State University Intramural Research Program, Columbus, OH, USA.

## Conflict of Interest

The authors declare that the research was conducted in the absence of any commercial or financial relationships that could be construed as a potential conflict of interest.

## Publisher’s Note

All claims expressed in this article are solely those of the authors and do not necessarily represent those of their affiliated organizations, or those of the publisher, the editors and the reviewers. Any product that may be evaluated in this article, or claim that may be made by its manufacturer, is not guaranteed or endorsed by the publisher.
