# Supplementary material for: Concomitant Medication Effects on Immune Checkpoint Inhibitor Efficacy and Toxicity
Source: Front Oncol. 2022 May 25;12:836934. doi: 10.3389/fonc.2022.836934 (PMC9196183; doi:10.3389/fonc.2022.836934)
Supplement: Supplementary file 1 [file DataSheet_1.pdf]

Supplementary Material

Concomitant Medication Effects on Immune Checkpoint Inhibitor Efficacy and Toxicity

Brendan Sieber[1], Julius Strauss[2], Zihai Li[3,4], Margaret E. Gatti-Mays[3,4]

[1] The Ohio State University, School of Medicine. Columbus, OH, USA.

[2] The Laboratory of Tumor Immunology and Biology, National Cancer Institute. National Institutes of Health. Bethesda, MD, USA.

[3]Division of Medical Oncology, The Ohio State University, Columbus, OH, USA.

[4]Pelotonia Institute for Immuno-Oncology, The Ohio State University, Columbus, OH, USA.

*** Correspondence:**
Margaret E. Gatti-Mays, The Ohio State University, 1800 Cannon Drive, 1300A Lincoln Tower, Columbus, OH, USA, 43210. Email: margaret.gatti-mays@osumc.edu

Keywords: immune checkpoint inhibitors (ICI); metformin; angiotensin converting enzyme inhibitor (ACEi); angiotensin receptor blockers (ARBs), aspirin

Supplemental Figure 1. Modified PRISMA Flow Diagram for Literature Review

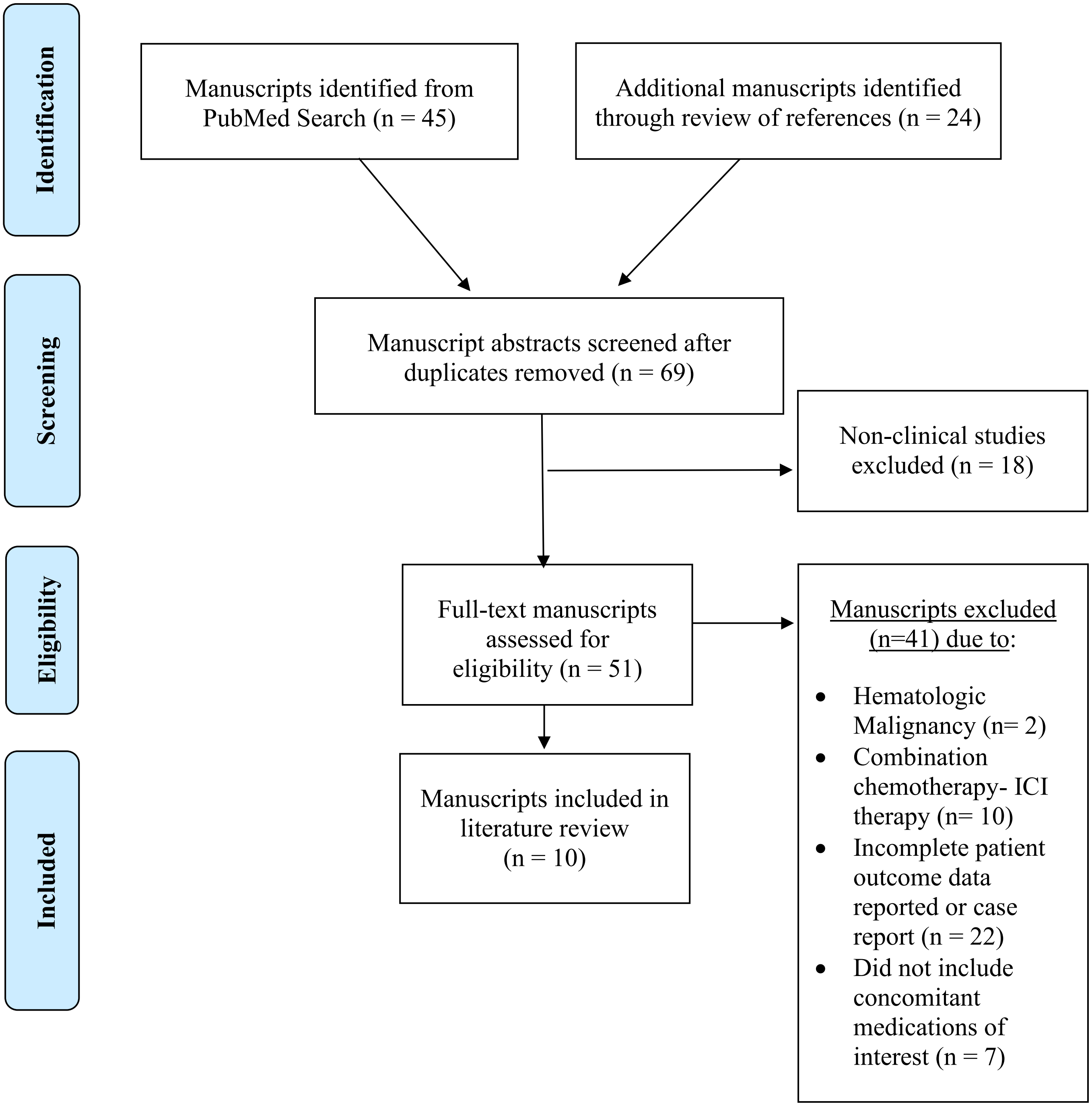

Supplementary Table 1: Expanded Summary of Impact of Concomitant Medications on ICI Efficacy and irAE Development

Study and Reference	Tumor type	ICI used	Median OS (months)	Median PFS (months)	ORR (%)	irAE
**Metformin**						
Afzal et al., 2018 (12)	Melanoma (n=55)	Ipilimumab, Nivolumab, and/or Pembrolizumab + Metformin (n=22)<br><br>Ipilimumab, Nivolumab, and/or Pembrolizumab (n=33)	↔<br>46.7 mon with<br>vs.<br>28 mon without<br><br>HR= 0.40 [95% CI = 0.12 to 1.35%]<br>p = 0.12	↔<br>19.8 mon with<br>vs.<br>5 mon without<br><br>HR = 0.55 [95% CI = 0.24 to 1.25%]<br>p =0.15	↔<br>68.2% with<br>vs.<br>54.5% without<br><br>p =0.31	↔<br>irAEs developed in<br>59% with vs 60.6% without p = 0.90<br><br>↑ pneumonitis 27.3% with vs 6.1% without p = 0.02

Afzal et al., 2019 (16)	NSCLC (n=50)	Pembrolizumab, Nivolumab, or Atezolizumab + Metformin (n=21)<br><br>Pembrolizumab, Nivolumab, or Atezolizumab (n=29)	↔<br>11.5 mon with<br>vs.<br>7.6 mon without<br><br>HR = 0.80 [95% CI = 0.39 to 1.63]<br>p=0.50	↔<br>4.0 mon with vs.<br>3.0 mon without<br><br>HR = 0.86 [95% CI = 0.47 to 1.6]<br>p=0.60	↔<br>41.4% with vs.<br>30.7% without<br><br>p=0.40	↔<br>irAEs developed in 38.1% with vs. 44.8% without p = 0.60<br><br>↓ pneumonitis 4.8% with vs 17.2% without p = 0.10
Cortellini et al., 2020 (33)	NSCLC (n=528)<br>Melanoma (n=263)<br>RCC (n=185)<br>Others (n = 36)	Pembrolizumab, Nivolumab, or Atezolizumab + Metformin use (n=114)<br><br>Pembrolizumab, Nivolumab, or Atezolizumab (n=898)	↔*<br>HR = 1.31<br>[95% CI = 1.02 to 1.70]<br>p=0.041<br><br>*aHR = 1.24<br>*(ECOG, metastatic burden, BMI)*<br>p = 0.10	↔<br>HR = 1.16<br>[95% CI = 0.92 to 1.47]<br>p= 0.19	↔<br>38.7% with vs.<br>37.5% without<br><br>OR = 1.06<br>[95% CI = 0.70 to 1.58]<br>p = 0.79	n/r

Cortellini et al., 2021 (30)	NSCLC (n=950)	Pembrolizumab + Metformin (n=125)<br><br>Pembrolizumab (n=825)	↔<br>HR = 1.14<br>[95% CI = 0.89 to 1.46]<br>p = 0.29	↔<br>HR = 1.03<br>[95% CI = 0.82 to 1.29]<br>p = 0.75	↔<br>OR = 0.83<br>[95% CI = 0.55 to 1.24]<br>p = 0.36	n/r
Failing et al., 2016 (29)	Melanoma (n=159)	Ipilimumab + Metformin (n=12)<br><br>Ipilimumab (n=147)	↔<br>HR 1.37<br>[95% CI = 0.48 to 3.88]<br>p = 0.56	↔<br>HR 1.83<br>[95% CI = 0.87 to 3.86]<br>p = 0.11	↔<br>18% with vs. 31% without<br><br>OR = 0.49<br>[95% CI = 0.10 to 2.43]<br>p = 0.38	↔<br>25% with vs. 30% without<br><br>OR = 0.83<br>[95% CI = 0.22 to 3.17]<br>p = 0.78
Gandhi et al., 2020 (31)	Melanoma (n=101)<br>Lung (n=68)<br>Renal (n=22)<br>Bladder (n=10)<br>Others (n = 9)	Nivolumab, Pembrolizumab, or Ipilimumab + Metformin (n=23)<br><br>Nivolumab, Pembrolizumab, or Ipilimumab (n=187)	↔<br>*Data not shown but reported as not significant*	↔<br>*Data not shown but reported as not significant*	n/r	n/r per medication

Gaucher et al., 2021 (32)	Lung (n=166)<br>Melanoma (n=110)<br>Genitourinary (n=27)<br>Head and neck (n=48)<br>Other (n = 21)	Ipilimumab, Nivolumab, Pembrolizumab, or Ipilimumab/Nivolumab + Metformin (n =17, Cohort A)<br><br>Ipilimumab, Nivolumab, Pembrolizumab, or Ipilimumab/Nivolumab (n = 355, Cohort B)	↔<br>HR = 0.77<br>[95% CI = 0.40 to 1.51]<br>p = 0.50	n/r	↑<br>47.1% with vs. 24.5% without<br>p = 0.020	n/r
Svaton et al., 2020 (27)	NSCLC (n=224)	Nivolumab + Metformin (n=18)<br><br>Nivolumab (n=206)	↔<br>10.6 mon with vs. 13.1 mon without<br><br>HR = n/r<br>p = 0.44	↔<br>3.3 mon with vs. 6.0 mon without<br><br>HR = n/r<br>p = 0.56	n/r	n/r
**ACEi/ARBS**						

Cortellini et al., 2020 (33)	NSCLC (n=528)<br>Melanoma (n=263)<br>RCC (n=185)<br>Others (n = 36)	Pembrolizumab, Nivolumab, or Atezolizumab + ACEi/ARBs (n=313)<br><br>Pembrolizumab, Nivolumab, or Atezolizumab (n=699)	↔<br>HR = 0.88<br>[95% CI = 0.72 to 1.07]<br>p = 0.22	↔<br>HR = 0.90<br>[95% CI = 0.76 to 1.07]<br>p = 0.24	↔*<br>42.9% with vs. 35.3% without<br><br>OR = 1.37 [95% CI = 1.04 to 1.82]<br>p = 0.03<br><br>*aOR = 1.26<br>[95% CI = 0.93 to 1.71]<br>p = 0.12	n/r
Failing et al., 2016 (29)	Melanoma (n=159)	Ipilimumab + ACEi/ARBs (n=30)<br><br>Ipilimumab (n=129)	↔<br>HR = 0.41 [95% CI = 0.10 to 1.71] p = 0.22	↔<br>HR = 0.67 [95% CI = 0.33 to 1.36] p = 0.27	↔<br>38% with vs.<br>28% without<br><br>OR = 2.01<br>[95% CI = 0.66 to 6.13]<br>p = 0.22	↔<br>35% with vs.<br>28% without<br><br>OR = 1.60<br>[95% CI = 0.57 to 4.46]<br>p = 0.37

Medjebar et al., 2020 (23)	NSCLC (n=168)	Pembrolizumab, Nivolumab or Durvalumab + ACEi (n=22)<br><br>Pembrolizumab, Nivolumab or Durvalumab (n=146)	↓*<br>9.82 mon with vs. 11.60 mon without<br><br>HR = 1.6<br>[95% CI = 1.0 to 2.7]<br>p = 0.07<br><br>*aHR = 2.0<br>*(ECOG, treatment line, BMI, comedication)*<br>p = 0.02	↓*<br>1.97 mon with vs. 2.56 mon without<br><br>HR = 1.8<br>[95% CI = 1.1 to 2.8]<br>p = 0.01<br><br>*aHR = 1.9<br>*(ECOG, treatment line, BMI, comedication)*<br>p = 0.01	n/r	n/r
Tozuka et al., 2021 (24)	NSCLC (n=256)	Nivolumab, Pembrolizumab, and Atezolizumab + ACEi/ARBs (n=37)<br><br>Nivolumab, Pembrolizumab, and Atezolizumab (n=219)	↔<br>22.6 mon with vs. 14.7 mon without<br><br>HR = 0.71<br>[95% CI = 0.45 to 1.11]<br>p = 0.13	↑<br>6.0 mon with vs. 2.2 mon without<br><br>HR = 0.59<br>[95% CI = 0.40 to 0.88]<br>p = 0.01	n/r	n/r

**Aspirin**						
Cortellini et al., 2020 (33)	NSCLC (n=528)<br>Melanoma (n=263)<br>RCC (n=185)<br>Others (n = 36)	Pembrolizumab, Nivolumab, or Atezolizumab + Aspirin use (n=189)<br><br>Pembrolizumab, Nivolumab, or Atezolizumab (n=823)	↔<br>HR = 0.94<br>[95%CI = 0.75 to 1.19]<br>p = 0.65<br><br>aHR = 0.85<br>*(ECOG, metastatic burden, BMI)*<br>p = 0.17	*↑<br>HR = 0.86<br>[95% CI = 0.71 to 1.06]<br>p = 0.16<br><br>*aHR = 0.79<br>*(ECOG, metastatic burden, BMI)*<br>p=0.03	*↑<br>44.4% with vs. 36.0% without<br><br>HR = 1.42<br>[95% CI = 1.02 to 1.97]<br>p = 0.04<br><br>*aHR = 1.47<br>*(ECOG, metastatic burden, BMI)*<br>p = 0.03	n/r
Cortellini et al., 2021 (30)	NSCLC (n=950)	Pembrolizumab + Aspirin (n=254)<br><br>Pembrolizumab (n=696)	↔<br>HR = 1.06<br>[95% CI = 0.87 to 1.28]<br>p = 0.53	↔<br>HR = 1.01<br>[95% CI = 0.85 to 1.20]<br>p = 0.89	↔<br>HR = 1.21<br>[95% CI = 0.89 to 1.63]<br>p = 0.22	n/r

Failing, et al., 2016 (24)	Melanoma (n=159)	Ipilimumab + Aspirin (n=38)<br><br>Ipilimumab (n=121)	↔<br>HR = 1.56<br>[95% CI = 0.79 to 3.10]<br>p = 0.20	↔<br>HR = 0.93<br>[95% CI = 0.55 to 1.57]<br>p = 0.79	↔<br>41.0% with vs. 27.0% without<br><br>OR = 1.87<br>[95% CI = 0.64 to 5.44]<br>p = 0.25	↔<br>41% with vs. 25% without<br><br>OR = 2.19<br>[95% CI = 0.85 to 5.64]<br>p = 0.10
Gandhi et al., 2020 (31)	Melanoma (n=101)<br>Lung (n=68)<br>Renal (n=22)<br>Bladder (n=10)<br>Others (n = 9)	Nivolumab, Pembrolizumab, or Ipilimumab + Aspirin use (n=58)<br><br>Nivolumab, Pembrolizumab, or Ipilimumab (n=152)	↔<br>*Data not shown but reported as not significant*	↔<br>*Data not shown but reported as not significant*	n/r	n/r per medication

↑ = statistically significant positive impact on ICI efficacy with concomitant medication use; ↓ = statistically significant negative impact with concomitant medication use, ↔ = no association between ICI effect/toxicity and concomitant medication use. * = adjusted ratio with significance. n/r = not reported.
